# Performance evaluation of Sanger sequencing for the diagnosis of primary hyperoxaluria and comparison with targeted next generation sequencing

**DOI:** 10.1002/mgg3.118

**Published:** 2014-11-05

**Authors:** Emma L Williams, Eleanor A L Bagg, Michael Mueller, Jana Vandrovcova, Timothy J Aitman, Gill Rumsby

**Affiliations:** 1 Clinical Biochemistry, Imperial College HealthcareLondon, United Kingdom; 2 Clinical Genome Informatics Facility, Department of Medicine, Imperial College LondonLondon, United Kingdom; 3 MRC Clinical Sciences Centre, Faculty of Medicine, Imperial College LondonLondon, United Kingdom; 4 Institute of Genetics and Molecular Medicine, University of EdinburghEdinburgh, United Kingdom; 5 Clinical Biochemistry, UCL HospitalsLondon, United Kingdom

**Keywords:** Primary Hyperoxaluria, genetic diagnosis, next generation sequencing, oxalate

## Abstract

Definitive diagnosis of primary hyperoxaluria (PH) currently utilizes sequential Sanger sequencing of the *AGXT, GRPHR*, and *HOGA1* genes but efficacy is unproven. This analysis is time-consuming, relatively expensive, and delays in diagnosis and inappropriate treatment can occur if not pursued early in the diagnostic work-up. We reviewed testing outcomes of Sanger sequencing in 200 consecutive patient samples referred for analysis. In addition, the Illumina Truseq custom amplicon system was evaluated for paralleled next-generation sequencing (NGS) of *AGXT*,*GRHPR*, and *HOGA1* in 90 known PH patients. *AGXT* sequencing was requested in all patients, permitting a diagnosis of PH1 in 50%. All remaining patients underwent targeted exon sequencing of *GRHPR* and *HOGA1* with 8% diagnosed with PH2 and 8% with PH3. Complete sequencing of both *GRHPR* and *HOGA1* was not requested in 25% of patients referred leaving their diagnosis in doubt. NGS analysis showed 98% agreement with Sanger sequencing and both approaches had 100% diagnostic specificity. Diagnostic sensitivity of Sanger sequencing was 98% and for NGS it was 97%. NGS has comparable diagnostic performance to Sanger sequencing for the diagnosis of PH and, if implemented, would screen for all forms of PH simultaneously ensuring prompt diagnosis at decreased cost.

## Introduction

The primary hyperoxalurias (PH) are autosomal recessive diseases (see Cochat and Rumsby 2013 for recent review) of glyoxylate metabolism characterized by excessive production of oxalate, which is excreted by the kidney. Calcium oxalate crystallizes in the urine, leading to nephrocalcinosis, urolithiasis, and consequent renal failure if treatment is not initiated promptly. The three known types of PH are PH1, due to mutations in the *AGXT* gene (Purdue et al. 1990), PH2, caused by mutations in *GRHPR* (Cramer et al. 1999), and PH3 arising from defects in *HOGA1* (Belostotsky et al. 2010). The different types of PH appear clinically very similar, with a similar age of onset (Williams et al. 2012) although treatment choices vary according to type, and it is, therefore, essential that an accurate diagnosis is established early in the course of disease to guide appropriate and effective treatment.

Diagnosis of PH currently relies upon stepwise Sanger sequencing of the exons and flanking intronic regions of the *AGXT, GRHPR*, and *HOGA1* genes (Cregeen et al. 2003; Williams and Rumsby 2007; Williams et al. 2012). This individual gene approach is costly and time-consuming and can contribute to delay in diagnosis, negatively impacting upon disease outcome (Hoppe and Langman 2003; van der Hoeven et al. 2012). Next generation sequencing (NGS) enables unrivaled sequencing capacity to be obtained at significantly reduced cost and faster turnaround time (Bockenhauer et al. 2012). Given these advantages, targeted NGS technologies are beginning to replace Sanger sequencing in diagnostic settings. However, it is essential to establish diagnostic validity of these approaches prior to their routine diagnostic use.

The University College London (UCL) Hospitals laboratory has offered a DNA sequencing service for the diagnosis of PH since 2006. PH1 testing comprises a step 1 test in which exons 1, 4, and 7 are sequenced, followed by analysis of the remaining exons (PH1 step 2) when step 1 does not identify two disease-causing mutations. Specimens testing negative for PH1, may progress to PH2 step 1 (exons 2 and 4 of *GRHPR*) and/or PH3 step 1 (exons 5 and 7 of *HOGA1*) followed by sequencing of the remaining exons of these genes. This approach allows the exons with the more common mutations to be sequenced first, enabling a more rapid diagnosis at reduced cost in some patients. Enzyme analysis is available for PH1 and PH2 testing and may be pursued in those patients where no mutations are detected, but requires a liver biopsy specimen.

Diagnostic accuracy of DNA testing for PH1 has been previously evaluated in liver biopsy proven patient cohorts, but the diagnostic yield of DNA sequencing for all three types of PH in the referral population has not been previously evaluated. In this manuscript, we review testing outcomes and diagnostic yield of Sanger sequencing of the *AGXT, GRHPR*, and *HOGA1* genes in 200 consecutive patient samples referred to the UCL Hospitals laboratory. In addition, we report an evaluation of the Illumina Truseq custom amplicon (TSCA) assay for simultaneous targeted NGS of the *AGXT*,*GRHPR*, and *HOGA1* genes in a retrospective analysis of 90 patients diagnosed with PH.

## Materials and Methods

### Samples

Blood and DNA samples from 200 patients with clinical symptoms suggestive of PH were referred for analysis. Informed consent for genetic testing was obtained by referring clinicians at time of patient work-up. The NGS evaluation utilized genomic DNA samples from 90 PH patients previously diagnosed by liver biopsy enzyme analysis (*n* = 59) or DNA analysis (*n* = 31), with genotypes determined by restriction digest analysis (Purdue et al. 1990; von Schnakenburg and Rumsby 1997) and/or Sanger sequencing (Cregeen et al. 2003; Williams and Rumsby 2007; Williams et al. 2012). Samples were anonymized prior to NGS and bioinformatics analysis was conducted by a researcher blinded to the initial patient diagnoses and genotypes obtained.

## Sanger sequencing

Genomic DNA was extracted from peripheral blood leukocytes using the QIAamp DNA Blood Mini Kit (Qiagen, Manchester, UK) according to manufacturer's instructions. *AGXT*,*GRHPR*, and *HOGA1* were each amplified in three large fragments by long-range polymerase chain reaction (PCR) using suitably positioned primers (Cregeen et al. 2003; Williams and Rumsby 2007; Williams et al. 2012). PCR products were cycle sequenced using internal primers and analyzed using an ABI 310 Genetic Analyzer (Applied Biosystems, Paisley, UK) as described previously (Williams et al. 2012).

## Evaluation of PH genetics service

Testing outcomes of Sanger sequencing were evaluated as the number of pathological variants found in each step of the analysis. The variants, their frequency, and exonic location were noted. Nomenclature is based upon the *AGXT, GRHPR*, and *HOGA1* cDNA sequences (Genbank Accession numbers NM_000030.2, NM_012203.1, and NM_138413.3, respectively), following HGVS recommendations (http://www.hgvs.org/), where nucleotide #1 is the first nucleotide of the ATG start codon. Pathogenicity of novel variants was assumed for splice-site and truncating mutations. For missense variants, pathogenicity was assigned based upon in silico prediction tools (SIFT, polyphen 2, and mutation taster), data from the 1000 genomes project and for PH1 modeling on the AGT crystal structure (Zhang et al. 2003). Diagnostic yield was assessed by determining the proportions of referred patients diagnosed with PH and via calculation of the posttest probability of disease.

## NGS targeted enrichment design

The TSCA kit (Illumina, San Diego, CA) was used to select for genes of interest, and to prepare amplicons for sequencing. The web-based Illumina DesignStudio was used to design a custom mixture of oligonucleotide probes, allowing sequencing of the genes of interest as defined by their chromosomal coordinates. Since sequencing of the full 3′-UTRs was not required, the 3′ coordinates of *AGXT* and *HOGA1* were truncated. The coordinates used in the design were as follows:

AGXT: 2:241808137 – 2:241818536
GRHPR: 9:37422707 – 9:37436986
HOGA1: 10:99344102 – 10:99371756

The DesignStudio software identified exonic regions and designed probes to generate amplicons using the following options: Exons only; amplicon length 250 bp; padding per exon: 25 bases; genome sequence: *H. sapiens* UCSC hg19 primary assembly. This process identified 42 amplicons covering 5242 bp, including the full coding regions and TCSA probes were synthesized by Illumina.

## NGS sample preparation and sequencing

DNA concentration was determined using the Quant-iT Broad-Range dsDNA Assay Kit (Invitrogen, Paisley, UK) measured with a Qubit 2.0 Fluorometer (Invitrogen). Quality and integrity were assessed by electrophoresis on a 0.8% agarose gel. Samples were prepared following the TSCA protocol using 250 ng of template DNA per reaction. Agencourt AMPure XP beads (Beckman Coulter, Inc., High Wycombe, UK) were used for PCR clean-up. Library normalization was performed according to the TSCA protocol, and compatible indexed samples were pooled.

The pooled library was diluted into HT1 buffer (Illumina) and spiked with PhiX (1%) prior to sequencing. Products were sequenced in multiplexed sequencing runs on the Illumina MiSeq generating 150 bp paired-end reads. Sequences were demultiplexed using MiSeq Reporter software allowing for one mismatch in the index sequence.

## NGS analysis

An initial test batch (*n* = 14) was analyzed to assess the performance of the protocol, then a further application batch with two patient samples from round one repeated, alongside 76 additional patient samples. In total samples from 64 PH1, 14 PH2, and 12 PH3 patients were analyzed, with one technical control and one negative control included per batch. Diagnostic sensitivity and specificity were determined and confidence intervals calculated using standard statistical procedures (Agresti and Coull 1998).

## NGS data analysis

Sequencing data from the MiSeq sequencer were exported as fastq files. Run parameters were assessed using the MiSeq Reporter and Amplicon Viewer applications (Illumina). Sequencing read quality was assessed with FastQC (http://www.bioinformatics.babraham.ac.uk/projects/fastqc). Reads were mapped to the GRCh37 (hg19) reference assembly with BWA version 0.6.1 (Li and Durbin 2010). Probe sequences were soft-clipped after alignment using custom Java code.

Indel realignment, quality score recalibration, and variant calling were carried out with the Genome Analysis Toolkit (GATK) version 2.6-5 (McKenna et al. 2010). Variants were called with the GATK Unified Genotyper algorithm considering only bases with a minimum base call quality score *Q*_phred_ of 20. Raw variant calls were hard filtered applying a coverage threshold of >30X and minor allele frequency of >15%. Functional annotation of variants was carried out with ANNOVAR (Wang et al. 2010) and SeattleSeq version 8 (http://snp.gs.washington.edu). The Integrative Genomics Viewer version 2.2 (Robinson et al. 2011) was used for visual inspection of read and variant data.

### Results

#### Evaluation of PH genetics service

Over a 5-year period, 200 patient samples were referred to the PH Genetics Service for diagnosis by Sanger sequencing. The mutations found are listed in Table1. PH1 step 1 analysis identified two mutations in 52 (26%) patients (Table1A). One mutation was found in 14 patients and in all cases the second mutation was detected by step 2 analysis (Table1B). All patients who were negative on step 1 analysis progressed to step 2 analysis, with two mutations detected in 34 patients (Table1C). Thus, step 2 analysis established a diagnosis in 48 (24%) patients.

**Table 1:** Outcomes of PH1 step 1 and step 2 testing in 200 consecutive samples referred for Sanger sequencing

Allele 1	Allele 2	No. of patients
(A) 2 mutations found in step 1 test		
c.508G>A	c.508G>A	19
c.33dupC	c.33dupC	8
c.508G>A	c.33dupC	5
c.508G>A	c.466G>A	3
c.447_454del8	c.447_454del8	2
c.731T>C	c.731T>C	2
c.508G>A	c.697C>T	1
c.508G>A	c.424-2A>G	1
c.508G>A	c.121G>A	1
c.508G>A	c.122G>A	1
c.508G>A	c.116_117dupCA	1
c.454T>A	c.116_117dupCA	1
c.454T>A	c.454T>A	1
c.454T>A	c.466G>A	1
c.33dupC	c.107G>A	1
c.33dupC	c.481G>A	1
c.77T>C	c.77T>C	1
c.473C>T	c.473C>T	1
c.725dupT	c.725dupT	1
(B) 1 mutation found in step 1 test, 1 mutation found in step 2 test		
c.33dupC	c.614C>T	1
c.33dupC	**c.866G>A**;**c.1076T>C**	1
c.121G>A	c.846+1G>A	1
c.454T>A	**c.353G>A**	1
c.508G>A	c.568G>A	1
c.508G>A	c.570delG	1
c.508G>A	c.614C>T	1
c.508G>A	c.653C>T	1
c.508G>A	c.777-1G>C	1
c.508G>A	c.834delC	1
c.508G>A	c.846+1G>T	2
c.508G>A	c.847-3C>G	1
c.508G>A	**c.1014C>G**	1
(C) 2 mutations found in step 2 test		
c.1049G>A	c.1049G>A	5
c.302T>C	c.302T>C	3
**c.519_520delinsGA**	**c.519_520delinsGA**	3
c.245G>A	c.245G>A	2
c.584T>G	c.584T>G	2
**c.596-2A>G**	**c.596-2A>G**	2
c.614C>T	c.614C>T	2
c.853G>T	c.853G>T	2
c.187G>C	c.187G>C	1
c.322T>C	c.322T>C	1
**c.324G>T**	**c.324G>T**	1
c.346G>A	c.346G>A	1
c.577dupC	c.577dupC	1
c.577dupC	c.847-3C>G	1
c.577delC	c.1079G>A	1
c.680+1G>C	c.680+1G>C	1
**c.777-2A>G**	**c.777-2A>G**	1
c.798_802del	c.798_802del	1
c.851T>C	c.851T>C	1
**c.922C>T**	**c.922C>T**	1
c.1007T>A	c.1007T>A	1

Novel variants are shown in bold. PH, primary hyperoxaluria.

Of the *AGXT* mutations detected, the exon 4 mutation c.508G>A represented 61 of 200 (31%) mutant alleles. The next most frequent mutation was the exon 1 mutation c.33dupC, found in 25 of 200 (13%) of mutant alleles. Of all mutant alleles identified, 46% were in exon 4 and 17% in exon 1, with only 3.5% in exon 7, the third exon analyzed in the step 1 test. In light of this finding, exon 7 sequencing has been removed from the PH1 step 1 test.

All 100 patients who tested negative for PH1 progressed to PH2 and PH3 step 1 testing, with the outcomes shown in Table2. Of these, 14 patients (7%) were found to have two PH2 mutations (Table2A) and 14 patients (7%) had two PH3 mutations (Table2B). One mutation was found in *GRHPR* in two patients and in *HOGA1* in two patients. In each case, the second mutation was detected by step 2 analysis. Therefore, in total 16 patients (8%) were diagnosed with PH2 and 16 patients (8%) were diagnosed with PH3.

**Table 2:** Outcomes of PH2 and PH3 step 1 and step 2 testing

Allele 1	Allele 2	No. of patients
(A) PH2 testing		
c.103delG	c.103delG	4
c.494G>A	c.494G>A	3
403_404+2delAAGT	403_404+2delAAGT	3
**84-8_84-5delCCCC; 84-13_84-12delCC**	**84-8_84-5delCCCC; 84-13_84-12delCC**	1
c.103delG	**c.288-2delAGT**	1
**c.102G>A**	**c.965T>C**	1
c.295C>T	**c.493**+**2T>A**	1
c.494G>A	**c.203T>C**	1
**c.743T>A**	**c.743T>A**	1
(B) PH3 testing		
c.700+5G>T	c.700+5G>T	8
c.107C>T	c.700+5G>T	1
c.107C>T	c.860G>T	1
**c.110G>T**	**c.953G>A**	1
c.700+5G>T	c.907C>T	1
c.700+5G>T	c.944_946delAGG	1
c.860G>T	c.944_946del AGG	1
c.875T>C	c.875T>C	1
**c.208C>T**	c.700+5G>T	1

All patients who tested negative for PH1 progressed to PH2 and PH3 Step 1 testing. PH2 step 2 and PH3 step 2 testing was performed in 43 and 32 patients, respectively, as requested by the referring clinicians. Novel variants are shown in bold. PH, primary hyperoxaluria.

Of those testing negative on the PH2 and PH3 step 1 tests, 27 patients progressed to PH2 step 2, of whom 16 also progressed to PH3 step 2. No further mutations were detected as a result of these analyses. Eleven patients progressed to liver biopsy analysis for alanine:glyoxylate aminotransferase and glyoxylate reductase activities, with PH1 and PH2 excluded in ten of these and PH1 confirmed in one. In this patient, exhaustive genetic analysis and family studies revealed a large *AGXT* gene deletion on one allele, but the other mutation was not detected.

The prevalence of the three forms of PH in the UCL Hospitals referral population has previously been estimated to be 0.63, 0.15, and 0.09, respectively (Williams et al. 2012). Prevalence of disease in the referral population equates to the pretest probability of disease. Sensitivities of the PH1 step 1 and PH1 step 2 tests were 65.3% (66 of 101 cases) and 99% (100 of 101 cases), respectively. Applying Bayes theorem, the finding of a pathological variant in PH1 step 1 or step 2 increases posttest probability of PH1 to 0.99. If no mutation is found in step 1, then the posttest probability is 0.37. If no mutation is found in step 2, the probability of disease falls to 0.017. Of the population previously referred for liver biopsy analysis, 3% were diagnosed with PH2, less than the 7% who tested positive by DNA analysis. This may reflect the fact that patients with suspected PH2 and increased L-glyceric acid excretion tended not to undergo liver biopsy analysis (Rumsby et al. 2004). Of 200 patient samples tested, 51 did not undergo complete sequencing of all three genes, leaving the diagnosis unresolved in 25% of patients referred.

### TruSeq custom amplicon targeted enrichment

**Figure 1:**
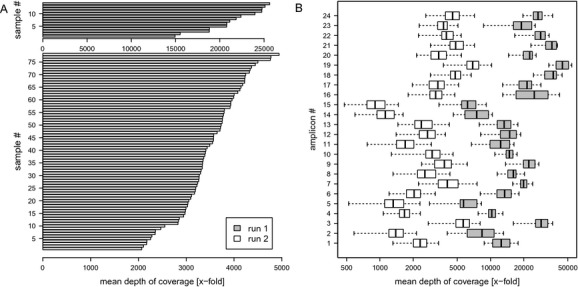
Depth of coverage. Amplicon coverage achieved when multiplexing 14 samples (run 1) or 78 samples (run 2) per MiSeq run. (A) Theoretical average coverage (calculated for each sample as total number of reads × read length/total target length). (B) Distribution of observed amplicon coverage after read mapping, clipping of probe sequences, and quality filtering.

To evaluate the TruSeq targeted enrichment assay, a total of 90 DNA samples were analyzed in two multiplexed sequencing runs on the Illumina MiSeq. In order to demonstrate scalability of the assay, 14 samples were sequenced in the first run and 78 in the second run. Altogether 23,338,572 150 bp reads were generated in the first run and 21,023,972 in the second run, equating to an average theoretical amplicon coverage of 24,268X and 3924X, respectively. After read mapping, clipping of probe sequences and quality filtering, the average depth of amplicon coverage was 21,092X and 3419X for samples in the first and second runs, respectively. The minimum average amplicon coverage was 6364X in the first run and 929X in the second, while the highest average amplicon coverage was 44,436X and 6927X, respectively (Fig.1). Two samples were sequenced in both runs allowing assessment of the impact of sequencing depth on variant detection. No differences were observed in the between-run base call and consequent variant detection for these two samples. Full exomic coverage of the genes of interest was achieved in both runs with sequence coverage extending to at least 55 bases upstream of the exons and at least 60 bases downstream.

The results of NGS analysis and previous DNA analysis are compared in Table3. Of 175 disease-causing alleles previously identified in the patient cohort, NGS analysis detected 174, the exception being a missense c.346G>A mutation in *AGXT* exon 3. The range of mutations for which the detection ability of NGS was tested included seven splice-site mutations (four in *AGXT*, two in *GRHPR*, and one in *HOGA1*), 16 indels (10 in *AGXT*, four in *GRHPR*, and two in *HOGA1*), and 39 missense/nonsense mutations (29 in *AGXT*, four in *GRHPR*, and six in *HOGA1*). These mutations included previously unpublished variants in *AGXT*; c.209C>A, c.215dupA, c.473delC, c.533G>A, c.996G>A, c.680+2T>A, c.847-1G>A and c.1045G>A, *GRHPR*; c.45delA, c.102G>A, c.493+2T>A, c.905G>A and c.965T>C and *HOGA1*; c.158delA.

**Table 3:** Comparison of the results of NGS and previous DNA analysis

Genotype by Sanger sequencing or restriction digest	Detected by NGS	Variant reads (%)	Variant effect	No. of patients
PH1 patients				
c.2_3delinsAT; c.33dupC	Yes; Yes	73;26	p.Met1Asn; p.Lys12fs	1
c.32_33delCC; **c.1045G>A**	Yes; Yes	30;62	p.Pro11fs; p.Gly349Ser	1
c.33dupC; c.33dupC	Yes; Yes	96	p.Lys12fs; p.Lys12fs	3
c.33dupC; c.454T>A	Yes; Yes	53;47	p.Lys12fs; p.Phe152Ile	1
c.33dupC; c.508G>A	Yes; Yes	52;29	p.Lys12fs; p.Gly170Arg	2
c.33dupC; c.547G>A	Yes; Yes	46;51	p.Lys12fs; p.Asp183Asn	1
c.33dupC; c.846G>C	Yes; Yes	44;56	p.Lys12fs; p.Gln282His	1
c.33dupC; c.886_888delGCG	Yes; Yes	48;49	p.Lys12fs; p.Ala296del	1
c.302T>C; c.302T>C	Yes; Yes	100	p.Leu101Pro; p.Leu101Pro	1
c.423G>T; c.423G>T	Yes; Yes	97	p.Glu141Asp; p.Glu141Asp	1
c.447_454delGCTGCTGT; c.447_454delGCTGCTGT;	Yes; Yes	100	p.Leu151fs; p.Leu151fs	2
c.481G>T; c.614C>T	Yes; Yes	25;53	p.Gly161Cys; p.Ser205X	1
c.508G>A; c.106C>T	Yes; Yes	49;41	p.Gly170Arg; p.Arg36Cys	1
c.508G>A; c.116_117dupCA	Yes; Yes^*^	44;99	p.Gly170Arg; p.Ala40fs	1
c.508G>A; c.121G>A	Yes; Yes^*^	46;95	p.Gly170Arg; p.Gly41Arg	2
c.508G>A; **c.209C>A**	Yes; Yes	49;54	p.Gly170Arg; p.Thr70Asn	1
c.508G>A; **c.215dupA**	Yes; Yes	45;56	p.Gly170Arg; p.Asn72fs	1
c.508G>A; c.322T>C	Yes; Yes	49;35	p.Gly170Arg; p.Trp108Arg	1
c.508G>A; c.454T>A	Yes; Yes	55;49	p.Gly170Arg; p.Phe152Ile	2
c.508G>A; c.508G>A	Yes; Yes	99	p.Gly170Arg; p.Gly170Arg	4
c.508G>A; **c.533G>A**	Yes; Yes	44;47	p.Gly170Arg; p.Cys178Tyr	1
c.508G>A; c.577dupC	Yes; Yes	46;47	p.Gly170Arg; p.Leu193fs	1
c.508G>A; c.653C>T	Yes; Yes	52;64	p.Gly170Arg; p.Ser218Leu	1
c.508G>A; c.697C>T	Yes; Yes	50;43	p.Gly170Arg; p.Arg233Cys	1
c.508G>A; c.731T>C	Yes; Yes	46;47	p.Gly170Arg; p.Ile244Thr	1
c.508G>A; c.738G>A	Yes; Yes	45;48	p.Gly170Arg; p.Trp246X	1
c.508G>A; c.847-3C>G	Yes; Yes	49;48	p.Gly170Arg; mis-splicing	2
c.508G>A; c.943-1G>T	Yes; Yes	46;52	p.Gly170Arg; mis-splicing	1
c.508G>A; **c.996G>A**	Yes; Yes	45;17	p.Gly170Arg; p.Trp332X	1
c.508G>A; unk	Yes; unk	49	p.Gly170Arg; unk	1
c.508G>A; **c.473delC**	Yes; Yes	57;40	p.Gly170Arg; p.Ser158fs	1
c.508G>A; **c.1076T>C**	Yes; Yes	47;57	p.Gly170Arg; p.Leu359Pro	1
c.560C>T; c.560C>T	Yes; Yes	100	p.Ser187Phe; p.Ser187Phe	1
c.577dupC; c.847-3C>G	Yes; Yes	51;47	p.Leu193fs; mis-splicing	1
c.568G>A; c.568G>A	Yes; Yes	99	p.Gly190Arg; p.Gly190Arg	2
c.584T>G; c.584T>G	Yes; Yes	100	p.Met195Arg; p.Met195Arg	1
c.661T>C; c.661T>C	Yes; Yes	98	p.Ser221Pro; p.Ser211Pro	1
**c.680+2T>A**; c.346G>A	Yes; **No**	58;10	Mis-splicing; p.Gly116Arg	1
c.697C>T; c.697C>T	Yes; Yes	100	p.Arg233Cys; p.Arg233Cys	1
c.698G>A; c.466G>A	Yes; Yes	55;80	p.Arg233His; p.Gly156Arg	1
c.698G>A; c.603C>A	Yes; Yes	55;53	p.Arg233His; p.Asp201Glu	1
c.731T>C; c.248A>G	Yes; Yes	48;54	p.Ile244Thr; p.His83Arg	1
c.731T>C, c.731T>C	Yes; Yes	99	p.Ile244Thr; p.Ile244Thr	3
c.731T>C; c.847-3C>G	Yes; Yes	45;54	p.Ile244Thr; mis-splicing	1
c.798_802 delinsACAATCTCAG; c.798_802 delinsACAATCTCAG	Yes; Yes	100	p.Ile267fs; p.Ile267fs	1
c.822G>C; c.822G>C	Yes; Yes	99	p.Glu247Asp; p.Glu247Asp	1
**c.847-1G>A**;**c.847-1G>A**	Yes; Yes	99	mis-splicing; mis-splicing	1
c.976delG; c.976delG	Yes; Yes	100	p.Val326fs; p.Val326fs	1
c.1049G>A; c.1049G>A	Yes; Yes	99	p.Gly350Asp; p.Gly350Asp	1
unk; unk	unk; unk		unk; unk	1
PH2 patients				
**c.102G>A**;**c.965T>C**	Yes; Yes	51;54	p.Trp34X; p.Met322Thr	1
c.103delG; c.103delG	Yes; Yes	100	p.Asp35fs; p.Asp35fs	2
c.103delG; c.84-2A>G	Yes; Yes	51;46	p.Asp35fs; mis-splicing	1
c.103delG; **c.905G>A**	Yes; Yes	100	p.Asp35fs; p.Arg302His	1
c.295C>T; **c.493+2T>A**	Yes; Yes	52;55	p.Arg99X; mis-splicing	1
c.403_404+2delAAGT; c.403_404+2delAAGT	Yes; Yes	100	mis-splicing	2
c.403_404+2delAAGT; c.540delT	Yes; Yes	26;34	mis-splicing	1
**c.45delA**;**c.45delA**	Yes; Yes	100	p.Ala17fs; p.Ala17fs	1
c.494G>A; c.494G>A	Yes; Yes	100	p.Gly165Asp; p.Gly165Asp	3
c.84-2A>G; c.84-2A>G	Yes; Yes	100	mis-splicing	1
PH3 patients				
c.107C>T; c.860G>T	Yes; Yes	49;16	p.Ala36Val; p.Gly287Val	1
c.346C>T; c.346C>T	Yes; Yes	96	p.Gln116X; p.Gln116X	1
c.700+5G>T; **c.158delA**	Yes; Yes	58;28	mis-splicing; p.Asp53fs	1
c.700+5G>T; c.208C>T	Yes; Yes	50;49	mis-splicing; p.Arg70X	1
c.700+5G>T; c.700+5G>T	Yes; Yes	100	mis-splicing	3
c.700+5G>T; c.907C>T	Yes; Yes	56;34	mis-splicing; p.Arg303Cys	1
c.860G>T; c.944_946delAGG	Yes; Yes	16;16	p.Gly287Val; p.Glu315del	1
c.875T>C; c.875T>C	Yes; Yes	100	p.Met292Thr; p.Met292Thr	1
c.944_946delAGG; c.944_946delAGG	Yes; Yes	100	p.Glu315del; p.Glu315del	2

For variants detected in multiple patients, the mean result is shown for % variant reads. The asterisk (^*^) indicates the two variants incorrectly assigned as homozygous by the NGS base calling. Novel variants are shown in bold. NGS, next generation sequencing; PH, primary hyperoxaluria.

NGS analysis was able to accurately determine mutation zygosity in the majority of cases, with exception of two *AGXT* mutations in exon one of the gene; c.116_117dupCA and c.121G>A, which were base called as homozygous although they were present in heterozygous state. Three patients diagnosed with PH1 were also heterozygous for the high-frequency *HOGA1* splice-site mutation c.700+5G>T.

There were no false positive diagnoses of disease; thus, the NGS assay showed 100% (95% CI ≥98%) diagnostic specificity. For PH1, diagnostic sensitivity was 97% (95% CI; 95–99) for Sanger sequencing and 95% (95% CI; 92–98) for NGS. For PH2 and PH3 both approaches had 100% (95% CI ≥98%) diagnostic sensitivity. The overall diagnostic sensitivity of Sanger sequencing was 98% (95% CI; 96–100). For NGS, it was 97% (95% CI; 95–99), giving the two sequencing approaches comparable diagnostic performance.

Minor allele status of *AGXT* could be assessed for all patients. This is particularly relevant for those with PH1 since several mutations, most notably c.508G>A, have increased pathogenicity when associated with the minor allele (see Williams et al. 2009 for review). Although it was originally reported that the c.32C>T, c.264C>T, and c.1020A>G variants are in complete linkage disequilibrium, it has since been established that the linkage breaks down in some cases, as reviewed in Williams et al. 2009. In our study, c.32C>T and c.264C>T were found in linkage disequilibrium in all homozygotes detected, but in five patients the copy number of c.1020A>G varied from that of the other two variants. Additionally, data from the 1000 genomes project reveal different minor allele frequencies (0.108, 0.109, and 0.137, respectively) for these variants, suggesting that the c.1020A>G variant is more frequent than c.32C>T and c.264C>T.

### Discussion

Our review of genetic testing outcomes of 200 consecutive patients' samples analyzed by Sanger sequencing found 50% diagnosed with PH1, 8% with PH2, and 8% with PH3. Of the patients referred, all three genes were sequenced in a further 8%, with no mutations detected. One patient with negative DNA results was diagnosed with PH1 by liver biopsy analysis. In the remaining 25% of patients, step 2 sequencing of the PH2 and PH3 genes was not requested, leaving their diagnosis unresolved. These findings prompted us to evaluate an NGS approach for simultaneous sequencing of all three genes within a single diagnostic test. This approach would ensure that all patients are screened for all three genetic causes of the disease early in their diagnostic work-up, enabling a more rapid diagnosis.

Genomic DNA samples from 90 PH patients were analyzed by NGS. Sanger sequencing of these samples had previously identified both disease-causing mutations in 88 patients. NGS analysis detected both disease-causing mutations in 87 of these. In the two remaining patients, both with liver biopsy proven PH1, neither approach detected two disease-causing mutations. Targeted NGS identified a variety of mutations including minor indels, but was not tested on its ability to pick up major deletions, relatively uncommon in these disorders. From analysis of other genes, including *CFTR* (Abou Tayoun et al. 2013) and *LDLR* (Vandrovcova et al. 2013) one might expect reduced copy number. The ability to detect large deletions could be assessed in future iterations of the assay design and with tailoring of the bioinformatics analysis.

NGS results showed 98% agreement with Sanger sequencing, with only four of 180 variants showing discrepant base calling. These exceptions all occurred in the *AGXT* gene and included a c.346G>A heterozygous change not identified by NGS in one patient. Interrogation of read mapping data revealed that this mutation was actually present in 10% of sequencing reads, suggesting that differential amplification of the two alleles in this region produced incorrect base calling. The other discrepancies occurred in three patients with mutations in exon one, where heterozygous variants were incorrectly called as homozygous; c.116_117dupCA in one patient and c.121G>A in two patients. These patients did not harbor any single nucleotide polymorphisms (SNPs) in the binding regions of the exon one sequencing probes, suggesting that preferential allele amplification was not the cause. Inspection of NGS read mapping data revealed a region of lower read depth spanning c.96-138, typically 40–60% of the surrounding regions, reflecting the fact that this area is only covered by one amplicon in the design. However, the read depth in this region of affected samples was over 400×, which should be sufficient for accurate variant calling. Future iterations of TSCA design should be altered to optimize coverage in these regions.

Targeted NGS showed comparable diagnostic performance to Sanger sequencing with sufficient test sensitivity and specificity for a clinical diagnostic assay. Similar diagnostic performance has been observed for other targeted NGS studies, including *CFTR* gene analysis for the diagnosis of cystic fibrosis (Abou Tayoun et al. 2013), *BRCA1* and *BRCA2* gene sequencing for hereditary breast and ovarian cancer (Feliubadaló et al. 2013), and a gene panel for the diagnosis of familial hypercholesterolemia (Vandrovcova et al. 2013). Targeted NGS analyses are not prone to difficulties currently encountered in whole genome sequencing (WGS), including areas of poor coverage, accuracy, and cost of after-sequencing bioinformatics analysis (reviewed in Chrystoja and Diamandis 2014). In addition, scalability of the assay has been demonstrated, enabling a diagnostic service to be provided without compromising cost or turnaround time. The availability of curated mutation databases for the three disorders (http://www.uclh.nhs.uk/phmd), would also assist with streamlined identification of pathological and nonpathological variants.

In all three known types of PH, there is usually significant hyperoxaluria, with excretion levels typically exceeding >0.7 mmol/1.73 m^2^ per 24 h (upper limit of normal 0.46). Not all patients with pronounced hyperoxaluria and high clinical suspicion of PH receive genetic testing. In the Netherlands, a retrospective review of 25 patients with elevated urine oxalate excretion identified one previously undiagnosed case of PH1 (van Woerden et al. 2007). A recent review of >5000 analyses performed in the UCL Hospitals laboratory revealed that genetic diagnosis of PH was not pursued in 24 patients, despite significantly elevated urine oxalate (Clifford-Mobley et al. 2014). Prompt diagnosis of PH is essential for timely treatment to preserve renal function, delay onset of renal failure, and limit oxalosis (Milliner 2005). There is, therefore, a clinical need for rapid and affordable screening for all forms of PH in stone-forming patients with grossly elevated oxalate excretion.

It is evident that delays in diagnosis of PH are common. In a survey of PH diagnosis, treatment and patient outcome in the United States fewer than half of patients underwent liver biopsy enzyme analysis for definitive diagnosis. Of those with a definitive diagnosis, 30% were diagnosed already in end-stage renal failure (Hoppe and Langman 2003). This picture is no different elsewhere; in a study of Dutch PH1 patients, 38% went undiagnosed until adulthood, 52% already in end-stage renal failure. Importantly, 68% of these patients were homozygous for the p.Gly170Arg mutation, which has a favorable prognosis when pyridoxine therapy is started early (Harambat et al. 2009; van der Hoeven et al. 2012).

This is the first study in which all three PH-causative genes have been analyzed in a large cohort of PH patients. Interestingly, three PH1 patients were heterozygous for the common *HOGA1* splice-site mutation c.700+5G>T. This is somewhat higher than would be expected, given the allele frequency was found to be only 0.09% in the 1000 genomes project. PH1 is known to be a clinically heterogeneous condition, which may in part arise from natural variation in other genes involved in related metabolic pathways. A previous study identified three idiopathic stone formers were heterozygous for variants in the *HOGA1* gene, including the c.860G>T mutation (Monico et al. 2011). *HOGA1* variants may possibly play a modifier role in disease expression in PH1 and this may become clearer as more patients have all three PH genes sequenced during their diagnostic work-up.

The identification of pathogenic variants in more than one of the genes associated with PH highlights one of the disadvantages of sequential analysis, wherein finding a single pathological mutation may lead one to incorrectly assume a diagnosis. A PH3 case has been reported in which the proband had a family history of PH1 and this patient was symptomatic for PH, despite carrying only one of the familial *AGXT* mutations. Full sequencing of the *AXGT* gene did not identify further likely PH1-causing variants, but subsequent analysis of *HOGA1* identified two disease-causing variants, leading to eventual diagnosis of PH3 (Beck et al. 2013).

In conclusion, targeted NGS analysis compared favorably with Sanger sequencing showing 98% agreement for the genotypes detected, with similar diagnostic performance. In two liver biopsy proven PH1 patients, neither Sanger sequencing nor NGS detected any mutations. Their disease may be due to promoter defects, or a result of sequence changes deep within introns leading to activation of cryptic splice sites. Since these regions are not covered by current sequencing approaches, in such cases liver biopsy enzyme analysis would still be required to confirm disease. Targeted NGS has potential to offer a cost-effective, scalable test for all three types of PH, enabling more rapid diagnosis than Sanger sequencing. NGS yielded additional information, including the simultaneous identification of variants in more than one of the PH genes. The implications of this finding for disease phenotype and patient prognosis may become clearer as routine genetic diagnostic testing embraces the next generation of DNA sequencing.
